# The inducible chemical-genetic fluorescent marker FAST outperforms classical fluorescent proteins in the quantitative reporting of bacterial biofilm dynamics

**DOI:** 10.1038/s41598-018-28643-z

**Published:** 2018-07-09

**Authors:** Amaury Monmeyran, Philippe Thomen, Hugo Jonquière, Franck Sureau, Chenge Li, Marie-Aude Plamont, Carine Douarche, Jean-François Casella, Arnaud Gautier, Nelly Henry

**Affiliations:** 1 Laboratoire Jean Perrin, CNRS UMR 8237 Sorbonne Université & UPMC Université Paris 06, F-75005 Paris, France; 2 PASTEUR, Département de Chimie, École Normale Supérieure, PSL University, Sorbonne Université, CNRS, 75005 Paris, France; 3 Laboratoire de Physique des Solides, CNRS, Université Paris-Sud, Université Paris-Saclay, 91405 Orsay Cedex, France; 4 Present Address: Institut de Physique de Nice, UMR 7010, Université Nice Sophia Antipolis, Nice, France

## Abstract

To increase our understanding of bacterial biofilm complexity, real- time quantitative analyses of the living community functions are required. To reach this goal, accurate fluorescent reporters are needed. In this paper, we used the classical fluorescent genetic reporters of the GFP family and demonstrated their limits in the context of a living biofilm. We showed that fluorescence signal saturated after only a few hours of growth and related this saturation to the reduction of oxygen concentration induced by bacterial consumption. This behaviour prevents the use of GFP-like fluorescent proteins for quantitative measurement in living biofilms. To overcome this limitation, we propose the use of a recently introduced small protein tag, FAST, which is fluorescent in the presence of an exogenously applied fluorogenic dye, enabling to avoid the oxygen sensitivity issue. We compared the ability of FAST to report on biofilm growth with that of GFP and mCherry, and demonstrated the superiority of the FAST:fluorogen probes for investigating dynamics in the complex environment of a living biofilm.

## Introduction

Bacterial biofilms are confined, three-dimensional communities of adherent microbes. They display an outperforming specific lifestyle, which raises a number of fundamental and practical questions drawing interest from various disciplines^1–6^. Molecular biology advances have pointed out hundreds of genes that are up- or down-regulated in biofilm-dwelling cells compared to their planktonic counterparts^7,8^. Moreover, no biofilm-specific gene has been identified so far^9^, suggesting that the key properties of biofilms might emerge from the subtle interplay between the unique physico-chemical environment of the cells within the biofilm and the elicited biological responses. In addition, a central feature of biofilms is their structural, chemical and biological heterogeneity at the microscale^10,11^, a property reflexively interrelated with the multiple gradients of nutrients, metabolites, oxygen and others molecules or ions present in a biofilm. Therefore, biofilm description relying on data averaging on the whole population is blurred and the clarification of the mechanisms behind the development of thriving adherent bacterial communities requires spatially-resolved approaches. Thus, biofilm quantitative descriptors of both gene expression and physico-chemical parameters should be probed with a spatial resolution allowing to take into account the biofilm heterogeneity and its implications for the biofilm growth. The advances in genetically encoded fluorescent proteins^12^ led to the development of fluorescence microscopy approaches for biofilm imaging^13^. Providing in principle molecular specificity, temporal and spatial resolution in living cells, the popular green fluorescent protein (GFP) and its variants have been broadly used for two decades now to monitor gene expression, label specific strains^14–20^, or measure specific functions in living biofilms^21–25^. However, quantitative analysis and studies in biofilms may be hampered by the sensitivity of the fluorescence of fluorescent proteins to oxygen level. Indeed, the final step of the chromophore maturation, which confers the ability to fluoresce to the protein, involves an oxygen-dependent dehydrogenation step requiring a minimal level of molecular oxygen^26,27^. Meanwhile, into biofilms, the spatio-temporal distribution of O_2_ which results from the balance of intermingled diffusion and consumption processes, is heterogeneous, poorly characterized and very dependent on the environmental conditions and the biofilm stage.

In this paper, we present the quantitative investigation of the spatio-temporal fluorescence profiles of two fluorescent proteins, GFP and mCherry, constitutively expressed in biofilms of *Escherichia coli* grown under controlled fluid flow. We show that both GFP and mCherry exhibit fluorescence kinetics quickly becoming inconsistent with the cell biomass development. By contrast, the spatio-temporal fluorescence profiles appeared to closely match the oxygen shortage that arises as the biofilm develops.

To circumvent this problem, we tested the ability of a new small protein tag, FAST (Fluorescence-Activating and absorption-Shifting Tag), to report linearly on gene expression in oxygen-deficient biofilms. Engineered by directed evolution, FAST holds its fluorescence from the dynamic and fully reversible binding of a small synthetic fluorogenic chromophore (so-called fluorogen)^28^. This process involves no oxidation step, allowing to anticipate no influence of the oxygen concentration on the fluorescence levels. By monitoring the spatio-temporal distribution of GFP and FAST fluorescence in the same biofilm growing under flow, we showed that the signal of the two proteins displays remarkably distinct spatial distributions and kinetics soon after biofilm starts to grow. Our findings also showed that in a mature biofilm FAST reports biomass spatial heterogeneity while GFP does not. Compared to micro-optical density measurements (*µ*OD), previously shown to linearly report biofilm growth in a limited range of values^29^, FAST fluorescence outperformed *µ*OD saturation, exhibiting an exponential increase well beyond *µ*OD signal inflexion. Using fluorogens with different spectral properties, we achieved biofilm labelling at distinct wavelengths and obtained similar results in experiments with the red fluorescent protein mCherry.

Altogether, our results demonstrate that the highly heterogeneous spatial and temporal distribution of oxygen in the biofilm strongly affects the fluorescence of classical GFP-like fluorescent proteins and therefore severely impaired the quantitative *in situ* measurement and interpretation of gene expression within living biofilms. The recently developed small protein tag, FAST^28^, which fluoresces in the presence of an exogenously applied fluorogenic dye, offers an outstanding answer to this issue, enabling accurate measurement of gene expression in biofilms, irrespectively of the oxygen gradients.

## Results

### Spatio-temporal distribution of GFP fluorescence does not linearly report on gene expression

**Figure 1:**
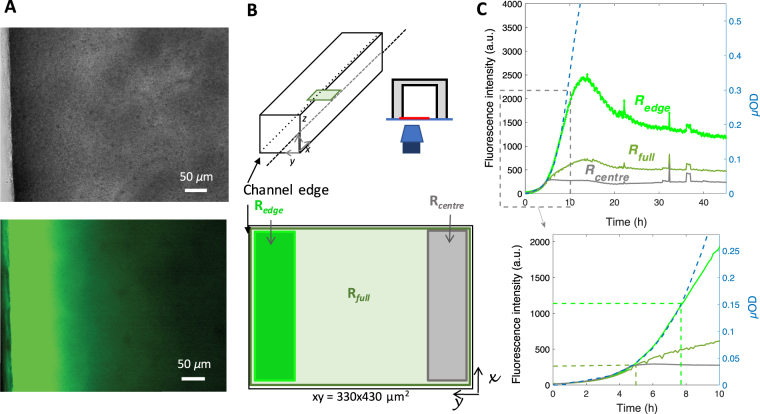
GFP fluorescence intensity in a living biofilm of *E. coli* displays unexpected spatial gradients and kinetics. (**A**) Brightfield (upper panel) and fluorescence (lower panel) images of an *E. coli* biofilm at the channel side wall vicinity after 24 hours growth under a continuous 1 ml/h nutrient flow. (**B**) Sketch of the square (1 mm × 1mm) PDMS channel, 3 cm length mounted on a microscope stage for real time imaging of biofilm light transmission and fluorescence. A 330 $\times$ 430 *µ*m^2^ field of view is imaged using a 20$\times$ objective. Defined regions of interest, R_*edge*_, R_*full*_ and R_*centre*_ are taken as shown in the bottom panel. (**C**) Plot of the fluorescence mean intensity per pixel kinetics (left axis) of the ROIs: R_*edge*_ (bright green), R_*full*_ (pale green) and R_*centre*_ (grey) optical density together with the microscopic optical density, *µ*OD = ln (I_0_/I) (right axis) of the ROI R_*full*_ (dashed blue line). Bottom panel shows a zoom of the first ten hours with the same colour code. For the sake of clarity, only the *µ*OD curve corresponding to a R_*full*_ type ROI is shown. *µ*OD curves from R_*edge*_ or R_*centre*_, essentially superimposable to R_*full*_ ones, are shown in Fig. S1 (Suppplementary Information) which also gives the statistical dispersion of fluorescence and *µ*OD curves coming from distinct experiments.

**Figure 2:**
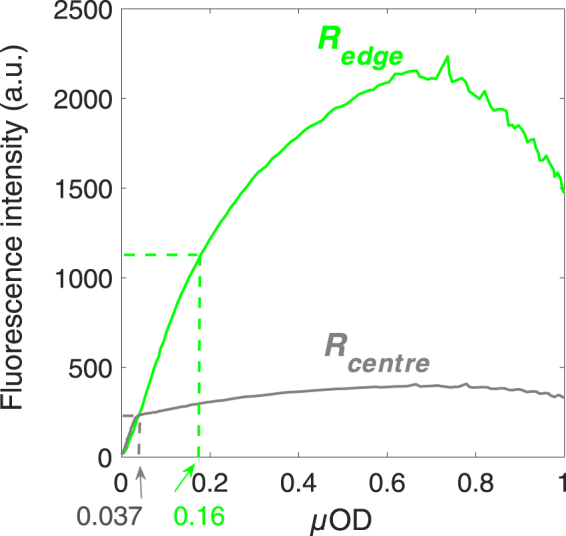
GFP fluorescence relation to biomass displays strong spatial heterogeneity. Fluorescence intensity is plotted as a function of the microscopic optical density, *µ*OD for the channel edge (bright green line) and centre (grey line) ROIs. Dashed lines indicate the fluorescence versus biomass linearity domains for the two ROIs and point to the corresponding *µ*OD values. The ROIs are defined as indicated in Fig. 1.

To quantitatively evaluate the spatio-temporal distribution of the most classical genetic reporter—*GFP*—in the confined environment of an adherent bacterial community, we grew a biofilm of *E. coli* cells constitutively expressing the pilus *F* that promotes biofilm formation and the fluorescent protein GFP (GFPmut3) in a PDMS channel continuously supplied with growth medium at a flow rate of 1 mL/h. The growing biofilm was monitored by collecting bright field and fluorescence images at a frequency of 12 images per hour (1 image every 5 min). As previously shown, the bright field images can be converted in microscopic optical density (*µ*OD) values, which linearly reported cell biomass^29^. GFP fluorescence intensity (I_*GFP*_) and *µ*OD were derived from the image stack reporting the first 24 hours of the biofilm growth. For each image (Fig. 1A), the mean values of intensity were averaged either over the whole field of view — R_*full*_ — or over smaller regions of interest (ROIs) located on the edge or in the centre of the channel, so called R_*edge*_ and R_*centre*_ as shown in Fig. 1B. Our first striking observation was that I_*GFP*_ and *µ*OD kinetics, which initially overlapped, strongly diverged after 5 hours of growth of the biofilm. While I_*GFP*_ curve saturated, *µ*OD kept on increasing (Figs 1C and S1). Interestingly, the I_*GFP*_ kinetics strongly depended, both in shape and intensity, on the localization of the ROI with respect to the channel edge (Figs 1C and S1). To better characterize this behavior, we plotted I_*GFP*_ as a function of *µ*OD (Fig. 2) for two ROIs, one at the edge and one in the centre of the channel. We observed that I_*GFP*_ deviation to linearity occurred for *µ*OD values decreasing by four-fold from the edge to the centre of the channel. Our observations indicated that above a given biofilm development point, the amount of fluorescent proteins synthesized by cells no longer matched the number of cells reported by the *µ*OD, contrarily to what is expected from a protein produced by a constitutively expressed gene. This discrepancy appears more strongly and quickly when the cells are further away from the edge of the channel while, under the hydrodynamical conditions used, no significant difference in biomass amount was measured between the edge and the centre of the channel. This suggested that an environmental parameter asymmetrically distributed in the channel affected the reporter fluorescence.

**Figure 3:**
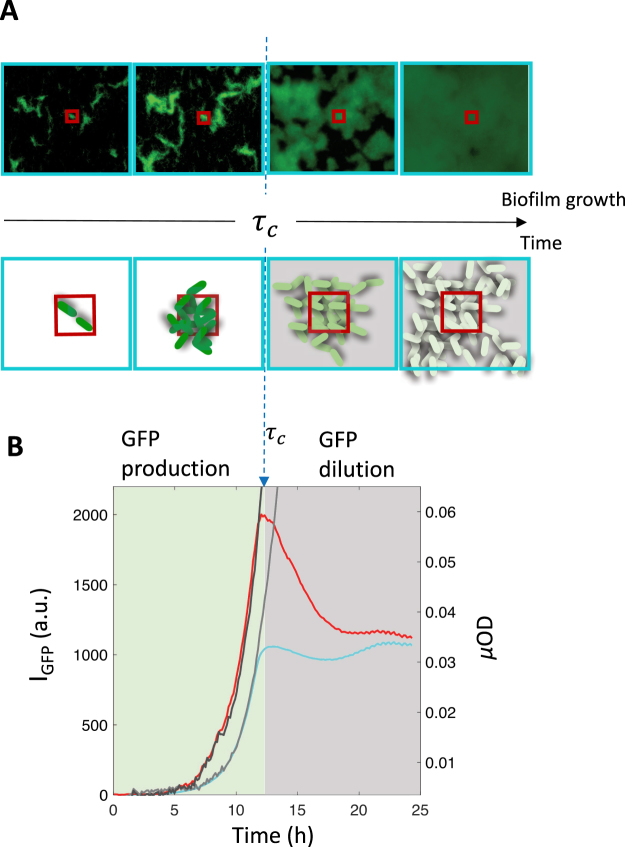
Biofilm growth kinetics displays a critical point where fluorescent protein production stops. (**A**) Images of a field of view, located in the middle of channel of an *E. coli* MG1655-*gfp*-F biofilm, taken at 6; 10; 16 and 20 hours after biofilm initiation (upper row) and showing both the cell fluorescence and the drawing of two types of ROIs of different size, the small one (red) and the larger one (cyan). The lower row sketches the principle of the small versus large ROI comparison — the small ROI being overpassed during the biofilm growth while the large one contains all the cells descending from the initial cluster lineage. (**B**) GFP fluorescence (left axis) kinetics is shown for the small (red curve) and large (cyan curve) ROIs together with *µ*OD signals (dark grey for the small ROI and light grey for the large ROI). The fluorescence intensity per pixel is averaged over the considered ROI. Data from different sets of ROIs are shown in supplementary Fig. S1 attesting the statistical accuracy of the observation.

To better understand the temporal variation of the fluorescence, we measured the evolution of fluorescence in two concentric ROIs of different sizes in the middle of the channel as shown in Fig. 3. The smaller ROI was drawn around an initial cluster of a few cells and the larger one contained a higher number of cells distributed over several clusters within the ROI. Then, we considered the mean fluorescence from each ROI, i.e. the total fluorescence divided by the number of pixels. Interestingly, the fluorescence intensity of the two ROIs displayed very distinct regime changes after about 12 hours of biofilm growth. The signal from the small ROI exhibited a steep fluorescence decrease while the one from the larger ROI remained essentially constant. In the meantime, the *µ*OD signal in the considered ROI increased exponentially, in agreement with the 3D growth of the biofilm. This behaviour indicated that, at the critical time ${\tau }_{c}$ of the fluorescence regime change, the mean fluorescence of single cells decreased while the total fluorescence of the cell population remained constant. In other words, after the time ${\tau }_{c}$, a dilution phase starts where new fluorescent proteins are no longer produced, the daughter cells sharing then fluorescent proteins synthesized in the initial regime (the production phase). So, the fluorescence of the small ROI decreased due to the spread of the initial cluster lineage beyond the limit of the ROI and the dilution of the fluorescent proteins, produced before the time ${\tau }_{c}$, out of the small ROI. Meanwhile the larger ROI, which contained the descendant cells of these initial clusters, displayed a constant global pool of fluorescent proteins although diluted within an increasing number of cells.

### GFP fluorescence in a growing biofilm is strongly impacted by the heterogeneous O2 concentration

At that stage, we made the hypothesis that the GFP fluorescence in the biofilm was strictly controlled by a threshold level of molecular oxygen below which the final oxidation step of the GFP chromophore maturation did not occur. We hypothesized that, when the biofilm grows and the cell oxygen consumption increases, the level of oxygen in the channel decreases below the threshold required for effective GFP fluorescence maturation. To test this hypothesis, we measured the spatial distribution of oxygen in a mature biofilm after approx. 20 hours of growth using micelles functionalized with fluorescent Ruthenium complex displaying oxygen-dependent fluorescence lifetime.

**Figure 4:**
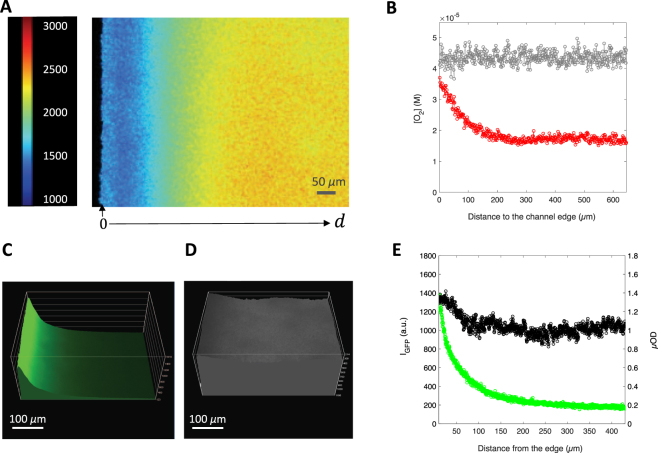
Within the living biofilm, the steep lateral gradient of GFP fluorescence matches the lateral gradient of oxygen concentration. (**A**) Fluorescence lifetime image of a mature biofilm of *E. coli* MG1655-*gfp*-F, grown during 20 hours, continuously supplied with a 1 ml/h medium flow containing Ruthenium micelles at a final Ruthenium concentration of 8 *µ*M. The field of view extends from the edge to the centre of the channel and captures 500 *µ*m of the channel length and captures a biofilm layer 7 ± 2 *µ*m thick close to the bottom of the channel. The colour bar is in nanoseconds. (**B**) Spatial molar oxygen concentration distribution as extracted from the lifetime image by averaging 3 contiguous lines of pixels for Ruthenium micelles alone (grey dots) or in the presence of the biofilm (red dots). (**C**,**D**) shows the spatial distribution of the GFP fluorescence (**C**) and *µ*OD (**D**) for an equivalent biofilm with the graph (**E**) of the values taken on each image along the same *y* line on these two images.

Biofilms were incubated with Ruthenium micelles and imaged by fluorescence lifetime microscopy (Fig. 4A) to derive oxygen spatial distribution (Fig. 4B) in the region extending from the edge to the centre of the channel. Control experiment performed in the absence of biofilm exhibited homogeneous O_2_ distribution in the whole channel (SI, Fig. S2). Then, we compared the O_2_ spatial distribution displayed in the presence of biofilm with that of I_*GFP*_ and *µ*OD measured under the same conditions (Fig. 4C,D). We found that I_*GFP*_ and O_2_ spatial distributions displayed similar profiles with both high fluorescence and high oxygen levels close to the edge of channel (Fig. 4B,E) and low levels at the centre of the channel. In the meantime, cell biomass displayed no significant gradient from the edge to the centre of the channel (Fig. 4D,E). The O_2_ gradient was consistent with the expected permeability of PDMS to oxygen. This was further confirmed by observing biofilms grown in glass channels with O_2_-impermeable edges, in which a uniform low level of fluorescence was observed in the channel (SI, Fig. S3).

Surprisingly, these results showed that after 20 hours of growth, colonization has expanded evenly over the channel, independently of the O_2_ access suggesting that the biofilm growth was not affected by O_2_ shortage. However, biomass transport and rearrangements during biofilm development could also account for this smooth topography.

These results emphasize the complexity of interpreting GFP fluorescence in a growing biofilm, due to the strong dependence of the fluorescence intensity on oxygen concentration, itself depending, in the biofilm, on local cell concentration and on the distance to the main oxygen sources in the considered devices, typically close to the edge of the channel for PDMS devices but also close to the channel connectors for devices made of materials with lower permeability to O_2_ such as for example glass or polystyrene^30^. Therefore, the fluorescence intensity is inconsistent with the biomass, unlike expected for a reporter under the control of a constitutive promoter. The O_2_ requirement for dehydrogenating the α, ß bond of the protein Tyr-66 residue during GFP fluorescence maturation has been pointed out early by Tsien, who indicated that the protein could not be used in obligate anaerobes^27^. Here, we show that GFP fails to fluoresce when the oxygen level of the environment drops below a threshold which is quickly achieved in a growing biofilm.

### mCherry fluorescence also exhibits a severe non-linearity in a growing biofilm

**Figure 5:**
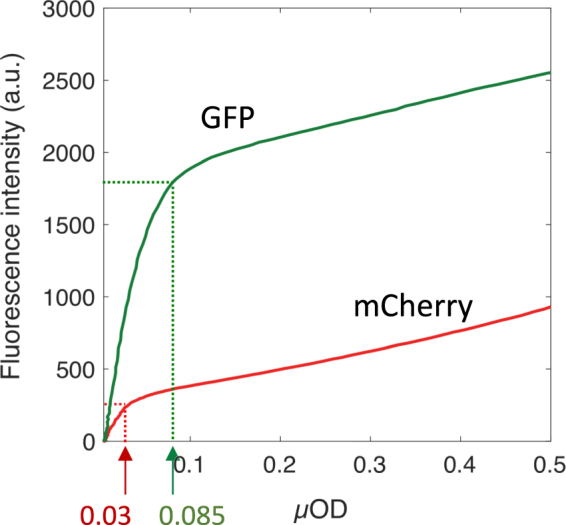
mCherry fluorescence saturates prior to GFP along the biofilm development. Variation of the fluorescence of mCherry versus GFP on parallel biofilms grown from *E. coli* MG1655-F-FAST-*mCherry* (red curve) and MG1655-*gfp*-F-FAST (green curve) under the same conditions. Fluorescence intensity per pixel is averaged over a central identical position in each channel. The dashed arrows indicate the fluorescence regime change and the corresponding values of *µ*OD.

To generalize our statement, we examined the fluorescence kinetics in a growing biofilm expressing the red fluorescent protein mCherry derived from *Discosoma sp*. Red (DsRed) fluorescent protein. As for GFP, mCherry expression was under the control of the promoter P_*R*_, an *E. coli* constitutive promoter, allowing to obtain expression that varies as cell biomass. In parallel channels, we initiated the growth of biofilms with cells expressing GFP and mCherry, respectively. We monitored fluorescence and *µ*OD signal kinetics along the biofilm growth and plotted for both reporters the variation of the fluorescence amplitude as a function of the *µ*OD derived from the same images (Fig. 5). We found that mCherry fluorescence also levelled off early in the biofilm development and that this saturation occurred for values of *µ*OD as low as 0.03 while GFP fluorescence remained strictly co-linear up to *µ*OD equal to 0.085. This result was in agreement with the larger sensitivity of mCherry to O_2_ shortage due to the two oxidation steps involved in the maturation of mCherry chromophore.

We concluded that fluorescent proteins relying on a O_2_-dependent chromophore maturation could not be used as quantitative tracers in living bacterial biofilms, in which the level of oxygen is low and heterogeneous.

### FAST outperforms GFP and mCherry in the complex environment of a living biofilm

**Figure 6:**
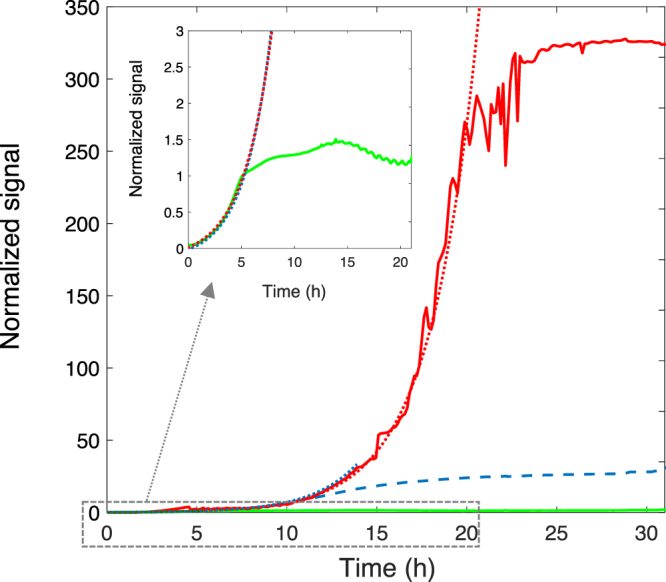
FAST-HBR-3,5-DOM, glowing in red, outperforms both GFP and *µ*OD signal in a living biofilm. GFP (green line) and FAST-HBR-3,5-DOM (red line) fluorescence kinetics shown together with *µ*OD (dashed blue line) signal all obtained from the analysis of a central ROI of the same biofilm of *E. coli* MG1655-*gfp*-F-FAST. All the curves have been normalized to unit at time t = 5 h. The adjustment of the experimental curves to exponential increases are shown as dotted lines of the same colour as the data lines. The insert shows a zoom of the GFP signal with the exponential adjustment of the *µ*OD and FAST-HBR-3,5-DOM kinetics.

To circumvent the oxygen dependence of classical fluorescent proteins, we tested FAST, a new fluorescent marker recently developed for labelling fusion proteins in living cells and organisms. FAST is a protein of 14 kDa engineered from the photoactive yellow protein (PYP) from *Halorhodospira halophila* to bind and activate the fluorescence of synthetic fluorogenic analogues of hydroxybenzylidene rhodanine (HBR). Because they fluoresce only when immobilized within FAST (because of conformational locking), HBR analogues do not exhibit nonspecific fluorescence in cells, enabling to detect FAST without the need for washing. FAST was shown to be functional in various expression systems including bacteria. As its fluorescence depends only on the interaction of the protein with its cognate fluorogen, FAST is fluorescent – provided that the fluorogen is present – whatever the oxygen level. In addition, an important feature of FAST is the ability to tune its emission colour from green-yellow to orange-red by changing the applied fluorogen, providing an experimental versatility not encountered with GFP-like fluorescent proteins. In a first set of experiments, we built a biofilm with MG1655-*gfp*-*F* cells transformed to constitutively express both GFP and FAST (see Materials and Methods). We supplied the fluorogen HBR-3,5-DOM at 2 *µ*M in the biofilm growth medium to form an orange-red fluorescent FAST detected in the ‘red’ channel (593/40 nm) while GFP fluorescence was collected in the ‘green’ channel (536/25 nm), having first checked that the presence of the fluorogenic dye did not altered bacteria growth rate (SI, Fig. S4) and that no background fluorescence was induced in the biofilm by the fluorogens in the absence of FAST expression (SI, Fig. S4). The expression of FAST and GFP, monitored in the same biofilm, displayed quite distinct fluorescence kinetics (Fig. 6). GFP signal saturated after approx. 5 hours growth while FAST fluorescence increased exponentially up to 20 hours. In between, *µ*OD signal levelled off after 13 hours of growth as expected due to the saturation of the detection for *µ*OD values above 0.5. Interestingly, within the first 5 hours of the biofilm development, the three signals increased exponentially with the same kinetic constant, 0.39 ± 0.02 h^−1^, showing parallel report of biomass and fluorescence over this initial time period (Fig. 6 insert).

**Figure 7:**
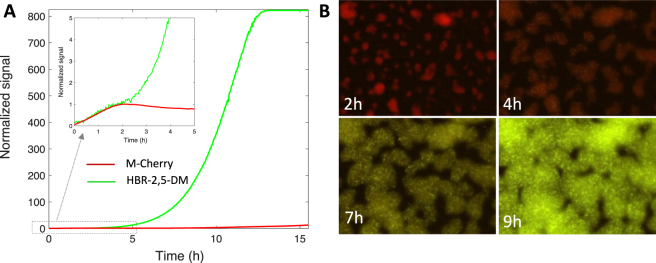
FAST, glowing in green with HBR-2,5-DM, early outperforms m-Cherry in a living biofilm. (**A**) mCherry (red line) and FAST-HBR-2,5-DM (green line) fluorescence intensity kinetics from a growing *E. coli* MG1655-F expressing the FAST-mCherry fusion protein. The signal on the graph has been normalized to unit at time t = 2 h which corresponds to mCherry saturation time. The insert shows a zoom in of the first five hours of the biofilm growth. (**B**) Overlay of mCherry and FAST fluorescence images collected of different times of the biofilm growth. Image acquisition time in each channel was chosen to capture the whole signal dynamics inducing a striking colour switch from the mCherry red emission, dominating the signal in the first 2 hours of the growth, to the yellow-green emission of FAST which largely overpasses mCherry’s after a few hours of growth.

We also examined the efficiency of the FAST protein in combination with another fluorogen, HBR-2,5-DM which emits in the green channel. We tested FAST:HBR-2,5-DM against mCherry, using MG1655-F cells transformed to constitutively express a mCherry-FAST fusion (see Materials and Methods). We grew biofilms in the presence of 2 *µ*M HBR-2,5-DM in the medium flow and we collected time-lapse fluorescence and *µ*OD images. mCherry and FAST fluorescence signals (Fig. 7A) diverged early during biofilm development as expected from the results displayed above regarding the dissimilarity between mCherry and GFP saturation stages (see Fig. 5). FAST:HBR-2,5-DM complex behaved quite similarly to FAST:HBR-3,5-DOM, increasing exponentially for several hours after mCherry saturation point. Fluorescence images acquired using exposure times enabling the capture of the whole FAST:HBR-2,5-DM fluorescence dynamics show the colour shift induced by mCherry saturation (Fig. 7B and movie S1) whereas FAST:fluorogen exhibits photophysical properties comparable to mCherry (Supplementary Information, Table S1).

We conclude that the FAST:fluorogen complexes are efficient and accurate reporters in the heterogeneous and low-O_2_ context of a growing biofilm.

**Figure 8:**
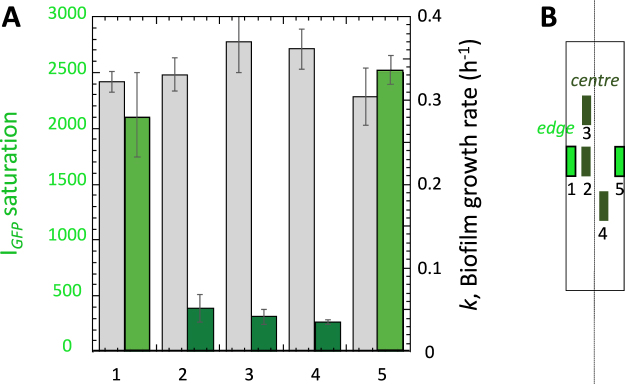
Biofilm growth rate is not affected by the local concentration of oxygen. (**A**) Biofilm growth rates derived from exponential adjustment of the FAST kinetics in an *E. coli* MG1655-*gfp*-F-FAST growing biofilm (grey bars, right axis) together with the saturation value of GFP fluorescence at the distinct channel locations. (**B**) Channel positions sketch.

As FAST enabled quantitative fluorescence measurements beyond the development limits imposed by *µ*OD signal saturation, it also provided a longer time scale to determine biofilm cell biomass growth rate. We made this analysis for several characteristic *edge* and *centre* regions using exponential adjustment of the FAST fluorescence intensity versus time curves, ${{I}_{{FAST}}={I}_{0}e}^{{kt}}$ to derive *k*, the biofilm growth rate. Figure 8 shows the plot of *k* for the different ROIs together with the saturation value of GFP at the same location which reflected the amplitude of the lack of O_2_ as shown above. Interestingly, we observed that O_2_ shortage which caused GFP fluorescence failure did not affect biofilm growth rate (Fig. 8). This means that the reduction of oxygen concentration, although blocking oxygen-sensitive reactions such as those controlling GFP or mCherry fluorescence, does not alter significantly biofilm growth.

## Discussion and Conclusions

Bacterial biofilm research currently goes through a rapid-fire expansion due to the diversity and the complexity of the questions raised by such a living community and its tremendous impact on our human lives and natural environment. Here, we focus our attention on real time optical imaging of biofilms with the aim of observing the dynamics of the adherent community *in situ* with a high spatio-temporal resolution. In this context, quantitative microscopy is essential and very promising, but raises the issue of accurate reporting. This study interrogated the use of genetically encoded fluorescent reporters of the GFP family to image living biofilm. We show that GFP and mCherry exhibit high sensitivity to the local level of oxygen. This oxygen sensitivity, which prevents the use of GFP-like fluorescent proteins under anaerobic conditions as originally reported by Tsien and co-workers, is experienced for an O_2_ level threshold rapidly reached under the concentrated and confined conditions of a growing biofilm, even under continuous flow. This leads to a spatial distribution of fluorescence intensities that depend on both the number of fluorescent cells and the local O_2_ concentration but also on the history of cells involving the number of cell divisions or biomass transport processes.

We show here results obtained with GFP and mCherry, although we also observed similar behaviours using YFP, Venus or mKate2 (data not shown). This O_2_-dependence is critical given that O_2_ distribution within a living bacterial biofilm depends a lot on the biofilm growth conditions such as the geometry of the setup, the O_2_ permeability of the device material, the flow rate which controls the amount of advected O_2_ but also all the conditions that will impact cell division rate and consequently cell consumption of O_2_ such as growth medium or temperature. The topography of the biofilm development also plays a role. Thus, oxygen distribution is hardly predictable in such complex systems. Nevertheless, considering the bacterial O_2_ consumption given in Riedel *et al*.^31^ for an exponentially growing population of *E. coli*, i.e. 1.7 × 10^6^ molec./s and an O_2_ medium concentration equal to 3 × 10^22^ molec./m^3^, we can calculate that the O_2_ contained in a cubic micro-volume of 100 µm side length in equilibrium with an atmospheric pressure of O_2_ or at the contact of a PDMS interface itself in equilibrium with atmosphere^32^, is fully consumed in less than 2 s by 10^4^ bacterial cells, which corresponds to a 20 µm thick layer of cells at a 20% volume fraction on a 100 µm side square. Besides, using an O_2_ diffusion coefficient in water of 3 × 10^−9^ m^2^/s, it can also be evaluated that the characteristic time for an O_2_ molecule to leave the 100 µm side cubic micro-volume is about 6 s. This emphasizes the high degree of O_2_ consumption by the bacteria in the confined environment of a growing biofilm, in agreement with the severe gradient of O_2_ observed in the channel for a mature biofilm. It is also worthwhile to consider the amount of O_2_ advected by the flow working at 1 ml/h. Using the same O_2_ concentration and consumption values as above, it can be shown that the 10^13^ molec./s contributed by the flow in the whole channel are fully consumed by a mature biofilm, underlining the micro-aerobic environment that should be expected in a millifluidic channel impermeable to gas such as a glass channel but also the key role of the oxygen diffusing by the channel wall in a PDMS device. However, it should also be kept in mind that bacterial oxygen consumption is expected to fluctuate a lot depending on many environmental and physiological parameters.

Therefore, quantitative interpretation of the fluorescence intensity of GFP and its homologs is not possible in a living biofilm. We propose here an alternative approach based on FAST, a small inducible chemical-genetic fluorescent marker composed of a protein part interacting reversibly with an exogenously applied fluorogenic chromophore that is non-fluorescent until labelling occurs. Fluorescence arises from the conformational locking of the fluorogenic chromophore within the binding cavity of FAST^28,33,34^. This unique fluorescence activation mechanism does not require molecular oxygen, making FAST a very good candidate for imaging proteins and cells in low-O_2_ environments. We demonstrate here that the FAST:fluorogen complexes efficiently labelled the biofilm, the small fluorogenic dyes diffusing freely and quickly in the biofilm. FAST allowed the reporting of exponential bacterial biofilm growth in flow at development stages where both GFP or mCherry fluorescence signal plateaued or decayed for hours. The ability to vary FAST colour changing the applied fluorogen opens exciting possibilities for various combinations with classical proteins. Indeed, beyond the measurement of a given gene expression *per se*, using the FAST system in combination with colour-complementing prototypical FP should allow real time monitoring of O_2_ variations in ratio mode. Moreover, assuming effectively that below a given O_2_ concentration threshold, no more fluorescent protein is produced and the cells are sharing the pre-existing pool could allow one to deduce the number of cell division number occurring locally by considering the FAST/FP fluorescence ratio to calculate the FP dilution factor.

Other families of non-prototypical genetically encoded proteins have emerged in the recent years^35^. Although O_2_-independent, due to their fluorogen-activated fluorescence, they show little irrigation of the microbe imaging field compared to the iconic GFP and none has been implemented in biofilm characterizations to the best of our knowledge. The LOV family proteins^36–38^ which fluoresce upon flavin binding detain photophysical properties, interesting for metallic ions or oxygen singlet biosensing but potentially restrictive for gene reporting, which possibly explains its limited spread. UnaG, a recently discovered protein^39^ from the Japanese eel, *Anguilla japonica*, fluoresces upon bilirubin binding in an O_2_-independent mechanism. Starting to be used in mammals as a hypoxia reporter and serum bilirubin sensor^40^, it might also offer an interesting tool for biofilm investigations.

The work we present here aims at stressing the absolute necessity to implement, in quantitative imaging studies dedicated to biofilms, fluorescent reporters displaying robust fluorescence whatever the local O_2_ level in order to be able to use the fluorescence signal as an accurate reporter of the protein expression level. We show that this is not the case for the classical FPs such as GFP because of their oxygen-dependent fluorescence and we propose an alternative method based on the system FAST that we successfully implemented in an *E. coli* biofilm. Further developments of multicolour orthogonal tag:fluorogen pairs should enable the study of multispecies biofilms.

## Materials and Methods

### Plasmids, strains and fluorogens

#### Plasmids

pAG87pR and pAG101pR were engineered from pAG87 and pAG101 described in Plamont *et al*.^28^ to replace T7 promoter with the lambda promoter p*R* for the expression of FAST and FAST-mCherry fusion in *E. coli* (details in *SI*). FAST is a variant of the photoactive yellow protein (PYP) containing the mutations C69G, Y94W, T95M, F96I, D97P, Y98T, Q99S, M100R, T101G^28^.

#### Strains

We used an *E. coli* MG1655-F strain carrying a non-conjugative F pilus plasmid to promote biofilm formation^41^ and its fluorescent variant, MG1655-*gfp*-F carrying *gfp-mut3* gene inserted on the chromosome under the control of the lambda-promoter p*R*. These strains were transformed with pAG101pR and pAG87p*R*, respectively, providing MG1655-F-FAST-*mCherry* which expressed the FAST-mCherry fusion protein and MG1655-*gfp*-F-FAST which expressed GFP and FAST proteins separately (details in *SI*).

#### Fluorogens

FAST fluorescence was activated using synthetic fluorogenic analogues of hydroxybenzylidene rhodanine (HBR) prepared as previously described^28,34^. We used HBR-2,5-DM (4-hydroxy-2,5-dimethylbenzylidene rhodanine) that fluoresces green light under blue light excitation when bound to FAST and HBR-3,5-DOM (4-hydroxy-3,5-dimethoxybenzylidene rhodanine), that fluoresces red light under green light excitation when bound to FAST.

### Microfabrication and biofilm

#### Millifluidic device

Millifluidic polydimethylsiloxane (PDMS) channels of 30 mm in length, 1 mm in width and height were micro-fabricated and bound to glass coverslips using oxygen plasma activation of the surfaces. Stainless steel connectors (0.013″ ID and 0.025″ OD) and microbore Tygon tubing (0.020″ ID and 0.06″ OD) supplied by Phymep (France) were used to connect fluid flow as described into more details previously^29^. The medium was pushed into the channels at a controlled rate using syringe pumps. When specifically required, we also used square glass capillaries, 800-*µ*m internal-side (Composite Metal Services, Shipley, UK).

#### Biofilm growth

All cultures were performed from LB agar plate colonies grown overnight in LB medium at 37 °C in an agitated Erlenmeyer flask in the presence of 7.5 *µ*g/ml tetracycline, then diluted in M63B1 minimal medium supplemented with 0.4% glucose to provide an exponentially growing culture with an OD_600_ equal to 0.2 after a few hours incubation at 37 °C. Biofilm growth was then initiated by injecting — approx. 3 × 10^6^ cells — of exponentially growing cells with an optical density at 600 nm equal to 0.2 in the channel and allowing static settlement for 90 min before starting culture medium flow at 1 ml/h and imaging bacterial development in the channel. The whole experiment was thermostated at 37 °C.

### Imaging

#### Microscope

We used an inverted NIKON TE300 microscope equipped with motorized x, y, z displacements and shutters. Images were collected using a 20× S plan Fluor objective, NA 0.45 WD 8.2–6.9. Bright field images were collected in direct illumination (no phase). Fluorescence acquisitions were performed using either the green channel filters for GFP and FAST:HBR-2,5-DM (Ex. 482/35, DM 506 Em. FF01-536/40) or the red ones for mCherry and FAST:HBR-3,5-DOM (Ex 531/40 nm DM 562 Em. 593/40). This last filter set corresponds to a compromise enabling to detect both HBR-3,5 and mCherry on the same optical path although not capturing the optimal excitation and emission wavelengths. Excitation was performed using a LEDs box (CoolLed pE 4000).

#### Image acquisition

We used a Hamamatsu ORCA-R2 EMCCD camera for time-lapse acquisitions of 1344 × 1024 pixels images with 12 bits grey level depth (4096 grey levels) and captured an *xy* field of view of 330 *µ*m × 430 *µ*m. Bright field and fluorescence images were usually collected for 24 hours at the frequency of 12 frames per hour. Excitation LEDs were set at a 50% power level and exposure times were 50 ms or 500 ms for the green emissions and 800 ms for the red emissions.

#### Image analysis

Image intensity per pixel averaged on defined regions of interest (ROIs) was collected using the NIKON proprietary software NIS. The data sheets edited by NIS were next exported to Matlab for further analysis of the biofilm development kinetics and growth parameters determination. Microscopic optical density (*µ*OD) was derived from transmitted light images according to *µ*OD = ln (*I*_0_/*I*), where *I*_*0*_ is the intensity (averaged gray level per pixel) recorded on a channel filled with medium only and *I* the intensity recorded on a channel containing a growing biofilm^29^, the presence of which attenuates incident light transmission. Fluorescence intensities were subtracted for their respective background using the contribution to the fluorescence intensity of a channel of medium in the absence of bacteria.

All curves are representative of three independent replicates. The statistical dispersion of the data is given in Fig. S1 where equivalent data coming from distinct positions and distinct experiments are shown. When numerical values are presented, they are given with a confidence interval which is the standard deviation of three equivalent measurements.

### Oxygen measurement

#### FLIM imaging

Lifetime images were acquired using a wide field frequency domain fluorescence lifetime imaging microscopy (FD-FLIM) setup, and a single plane illumination (SPIM) obtained using a beam expander and a cylindrical lens 45 mm of focal length. The thickness of the illuminated plane was determined to be 7 ± 2 *µ*m in the object plane of the microscope. The setup was equipped with a 445 nm laser diode module and an intensified CCD camera that can both be modulated synchronously from 0.1 to 200 MHz. Fluorescence lifetime was derived from the phase shift and relative modulation of the fluorescence sample when compared with a reference sample of known lifetime — here a hypericin solution, 3 ns lifetime.

#### Oxygen probe

Dissolved O_2_ was measured by quenching a Ruthenium complex (Ruthenium–tris(4,7- diphenyl-1,10-phenanthroline) dichloride (Ru(dpp)); Fluka) encapsulated in micelles of (1,2-Dipalmitoyl–sn-Glycero-3-Phosphoethanolamine-N[Methoxy(Poly-ethylene glycol)-2000] (DMPC-PEG2000) phospholipids; Avanti. The encapsulation of the Ru(dpp) complex in the micelles^42^ yields it biocompatible whereas free Ru(dpp) is strongly toxic for cells. Ru–micelles were added to the millifluidic channels with or without biofilm at a concentration of 8 μM for fluorescence lifetime imaging. Oxygen level was converted from fluorescence lifetime values using the Stern-Volmer relation^43^ and values of the quenching constant and of the zero-oxygen lifetime equal to 4.33 × 10^4^ M^−1^ and 3809 ns, respectively.

## Electronic supplementary material

Movie S1

Supplementary Information
