# Supplementary material for: The inducible chemical-genetic fluorescent marker FAST outperforms classical fluorescent proteins in the quantitative reporting of bacterial biofilm dynamics
Source: Sci Rep. 2018 Jul 9;8:10336. doi: 10.1038/s41598-018-28643-z (PMC6037777; doi:10.1038/s41598-018-28643-z)
Supplement: Supplementary file 2 — Supplementary Information [file 41598_2018_28643_MOESM2_ESM.docx]

# The inducible chemical-genetic fluorescent marker FAST outperforms classical fluorescent proteins in the quantitative reporting of bacterial biofilm dynamics

Amaury Monmeyran^1^, Philippe Thomen^1^, Hugo Jonquière^1^, Franck Sureau^1^, Chenge Li^2^, Marie-Aude Plamont^2^, Carine Douarche^3^, Jean-François Casella^1^, Arnaud Gautier^2^ and Nelly Henry^1^

^1^ Sorbonne Université, UPMC Univ Paris 06 & CNRS, UMR 8237, Laboratoire Jean Perrin, F-75005, Paris, France

^2^ PASTEUR, Département de Chimie, École Normale Supérieure, PSL University, Sorbonne Université, CNRS, 75005 Paris, France.

^3^Laboratoire de Physique des Solides, CNRS, Université Paris-Sud, Université Paris-Saclay, 91405 Orsay Cedex, France

Supplementary Information

**Plasmids:**

pAG87pR and pAG101pR were engineered from pAG87 and pAG101 — carrying respectively FAST gene and FAST-mCherry gene fusion under the control of T7 promoter with a kanamycin resistance1 as follows: T7 promoter was replaced by pR promoter digesting plasmid vectors with BglII and XbaI (Fast Digest enzymes, Fermentas) and purifying them on agarose gel. The pR promoter sequence was obtained by hybridizing two DNA oligonuleotides sequences (Eurofins): GATCGACTATTTTACCTCTGGCGGTGATAATGGTTGCATGTACTAAGGAGGTTG

and

CTAGCAACCTCCTTAGTACATGCAACCATTATCACCGCCAGAGGTAAAATAGTC

The digested and purified plasmids were mixed with hybridized oligonuleotides and ligated using a DNA Ligation Kit (ThermoFisher). Escherichia coli DH5$\alpha$ strain was prepared to be heat shock transformed with the ligation product, then streaked on kanamycin selective plates. After overnight growth, the colonies were screened by adding HBR-3,5 DOM on plates and observing colonies fluorescence under the microscope. Fluorescent clones were recovered. The sequence of each clone was checked by DNA sequencing before plasmid extraction. MG1655-F and MG1655-gfp-F strains were prepared to be heat shock transformed with pAG101pR and pAG87pR, respectively.

[1] Plamont, M. A., Billon-Denis, E., Maurin, S., Gauron, C., Pimenta, F. M., Specht, C. G., Shi, J., Querard, J., Pan, B., Rossignol, J., Morellet, N., Volovitch, M., Lescop, E., Chen, Y., Triller, A., Vriz, S., Le Saux, T., Jullien, L., and Gautier, A. (2016) Small fluorescence-activating and absorption-shifting tag for tunable protein imaging in vivo, Proc Natl Acad Sci U S A 113, 497-502.

**Supplementary Figures**

**Figure S1: *µ*OD and fluorescence signals display very distinct dependence on their location in the channel.** (A) *µ*OD and (B) GFP fluorescence kinetics are shown for *edge* (cyan in A and light green in B) and *centre* (dark blue in A and dark green in B) ROIs from three different channels defined by their line stroke (solid, dashed, dotted). It appears that *µ*OD signal (A) which reports biomass up to values of approx. 0.5 does not significantly depend on the reported position — edge or centre. In this case, the bundle of curves only gives the statistical dispersion of the measurements for distinct experiments. Indeed, no larger difference is observed between equivalent positions from distinct or same channel and edge or centre positions from the same channel. By contrast, GFP fluorescence curve collection exhibits two distinct bundles of curves clearly depending on the reported position — edge or centre. This difference largely overpasses the experimental dispersion observed for equivalent positions coming from different channels, same line stroke as for A.

**Figure S2: O_2_ spatial distribution in the channel in the absence of biofilm.** (A) Fluorescence lifetime image of medium containing Ruthenium micelles at a final Ruthenium concentration of 8 *µ*M. The field of view extends from the edge to the centre of the channel and captures 500 *µ*m of the channel length. The image shows a uniform distribution of O_2_ in the channel with an average lifetime value of $1324\pm64 ns$ which reported an O_2_ concentration of $(4.34\pm0.3)\times{10}^{-5}$M, consistent with atmospheric oxygen equilibrium in water. (B) Lifetime profiles taken at different *y* positions in the channel — superimposable and flat they confirm the uniform O_2_ distribution reported by the Ruthenium micelles in the absence of O_2_ depletion by the biofilm.


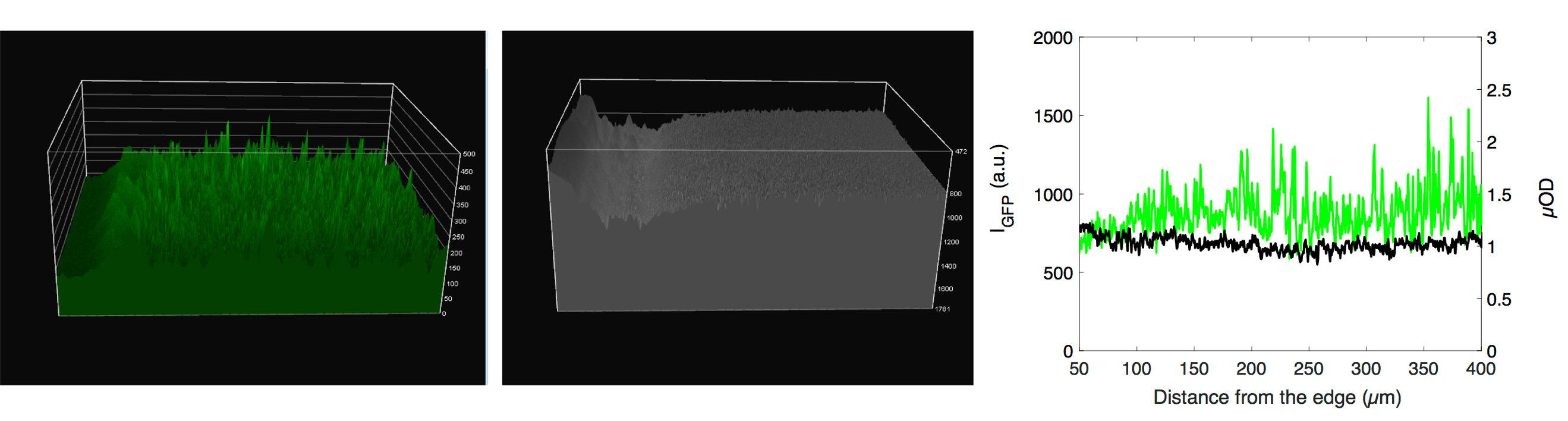


**Figure S3: GFP fluorescence displays even spatial distribution in a glass channel.** (A)Three-dimensional distribution of GFP fluorescence (left image) and transmitted light intensity (right image) in a mature biofilm of *E. coli* MG1655-*gfp*-F, grown during 20 hours, continuously supplied with a 1ml/h medium flow. (B) Graph of the two signals according to a transversal cross-section from the channel edge to the channel centre, the first 50 µm close the channel wall are not represented due to the optical perturbations generated by the glass wall thickness. In the glass channel the fluorescence intensity is evenly low due the absence of oxygen supply through the side walls by contrast with PDMS channels.

**Figure S4: GFP Fluorogens do not affect bacterial growth at the concentration used in experiments and up to 20 µM**.

Overnight culture of *E. coli* was diluted in LB medium supplemented or not with fluorogen HBR-2.5-DM or HBR-3,5-DOM, at 2 or 20 µM in a multiwell plate. Cell growth was measured for all samples using a TECAN plate reader at 37°C with agitation for 24 hours. The growth curves were adjusted to exponential functions providing the generation time for each condition which were monitored in triplicate. Histograms indicate the mean value of the generation time, and bars indicate standard error.

**Figure S5: Fluorogens alone do not contribute to fluorescence background.** Fluorescence intensity of a biofilm grown for 16 hours from *E. coli* MG1655-F strain in the presence (solid lines) or in the absence (dotted lines) of 2µM of HBR-2,5-DM (green curves) or HBR-3,5-DOM (red curves). The dotted curves from biofilm grown in the absence of fluorogen appear in green when detected in the GFP optical path and in red when detected in the mCherry optical path. The corresponding *µ*OD curves are shown in light blue (… , no fluorogen; --- , HBR-2,5-DM; -.- , HBR-3,5-DOM). The data show that the presence of the fluorogens does not bring about any additional contribution to the background biofilm fluorescence. Typical curves obtained on similar biofilms grown from strains expressing FAST are displayed for the sake of comparison (dashed lines, green for HBR-2,5-DM and red for HBR-3,5-DOM).

**Supplementary Table**

|  | λ_abs_ (nm) | λ_em_ (nm) | ε (M^–1^.cm^–1^) |  | Brightness |
| --- | --- | --- | --- | --- | --- |
| GFP | 488 | 507 | 56000 | 0.60 | 34 |
| mCherry | 587 | 610 | 72000 | 0.22 | 16 |
| FAST:HBR-2,5-DM | 494 | 552 | 50000 | 0.29 | 14.5 |
| FAST:HBR-3,5-DOM | 518 | 600 | 39000 | 0.31 | 12 |

**Table S1: Proteins and fluorogens photophyical properties.** Excitation and emission wavelength (λ_abs_ and λ_em_), molecular extinction coefficient (ε), quantum yield ($)$ and Brightness.

**Supplementary Movie**

**Movie S1:** **Biofilm growth imaging over the first 12 hours of growth**. MG1655-F-FAST-*mCherry* was grown in the presence of HBR-2,5-DM 2*µ*M and mCherry (red channel) and HBR-2,5-DM (green channel) stacks of images have collected in parallel using a 400 ms and 100 ms acquisition time at a frequency of one frame very 4 mins and overlaid. Other details as in Fig. 4.
